# Sustainability in Dentistry: Assessing knowledge, attitude, and practices of dental practitioners about green dentistry

**DOI:** 10.12669/pjms.40.1.7606

**Published:** 2024

**Authors:** Nighat Zia, Jennifer Geraldine Doss, Jacob John, Jeneen Panezai

**Affiliations:** 1 Nighat Zia PhD Student, Department of Community Oral Health & Clinical Prevention, Faculty of Dentistry Universiti Malaya, 50603 Kuala Lampur, Malaysia; 2 Jennifer Geraldine Doss Professor, Dept. of Community Oral Health & Clinical Prevention, Faculty of Dentistry Universiti Malaya, 50603 Kuala Lampur, Malaysia; 3 Jacob John Associate Professor, Department of Restorative Dentistry, Faculty of Dentistry Universiti Malaya, 50603 Kuala Lampur, Malaysia.; 4 Jeneen Panezai Assistant Professor, Faculty of Life Sciences and Informatics, Baluchistan University of Information Technology, Engineering and Management Sciences, Quetta, Pakistan

**Keywords:** Eco-friendly dentistry, Green dentistry, Sustainable dentistry, Knowledge, Attitudes, Practice

## Abstract

**Objective:**

Green dentistry is an emerging concept necessary to address the worsening climatic changes. It is essential to compile the existing literature on knowledge, attitude, and practice on green dentistry that can be accomplished by conducting a literature review. The objective of this literature review was to summarize and present the existing knowledge that dentists have regarding green dental practices, their attitude about this shift towards sustainability, and steps that they have taken in their personal practice to adhere towards an eco-friendlier dental approach.

**Methods:**

Three months of effective research and review development from March 2022 to June 2022. Design using keywords, a literature search was performed in PubMed, Google scholar and Web of Science databases. A total of 13 articles of 45, fulfilling the inclusion criteria were selected, of which two were excluded as these were not in English.

**Results:**

Dental practitioners have good knowledge about green dentistry and positive attitudes towards environment conservation, but implementation in their practice is not adequate. Most common themes of knowledge, attitude and practice assessment in green dentistry are amalgam management, radiographic management, infection control, waste management, water, and electricity management.

**Conclusion:**

The absence of adequate literature on eco-friendly practices in dentistry makes it difficult to validate the findings of most of these studies. Dental professionals are familiar with environmentally friendly dental practices and have a positive outlook on their role in environmental protection, but its application in practice is far from adequate.

## INTRODUCTION

Sustainable Development Goals (SDGs) were presented in the United Nations (UN) in 2015, by encouraging all countries to adopt a set of 17 goals targeted to end poverty, protect the planet, and ensure prosperity for all. Sustainable development is becoming increasingly important due to environmental concerns.^1^ Climate change is caused by human actions that produce greenhouse gases, leading to rising temperatures, heat waves, fires, droughts, and warming waters.^2^ The healthcare sector is committed to providing safe and effective care.^3^ Health and dental care providers prioritize efficiency over sustainability, leading to the production of a lot of waste and carbon dioxide emissions.^4^

Dental practices discard millions of sterilization pouches, chair barriers, and light handle covers annually. Gloves, masks, suction tips, saliva ejectors, needles, and paper as other disposables are difficult to breakdown.^5^ 360 gallons of water waste each day, production of 4.8 million lead foils, 28 million liters of poisonous X-ray fixer, and 3.7 tons of mercury waste annually.^5^ Farahani and Suchak pioneered eco-friendly dentistry in 2007. His recommendations have been heavily used in subsequent studies.^5^ The World Federation of Dentistry (FDI) called for sustainable techniques in oral health care to safeguard service delivery and foster green economy.^6^

Since the idea of “green dentistry” is novel, only a few studies have explored dentists’ knowledge, attitudes, and practices (KAP) in order to learn more about what they know, believe, and engage in.^7^ However, these studies are not unidirectional and cover various aspects of KAP of dentists with regards to sustainable dental practices. Compiling and analyzing existing KAP literature is essential to identify gaps and guide future research efforts. KAP questionnaires can be used to inform further research. Literature review was identified as the most effective method. This literature review aims to summarize the knowledge and attitudes of dentists regarding sustainable dental practices, their attitude towards this shift, and their steps to adhere to an eco-friendly dental approach.

## METHODS

### Literature search Design

Literature search and review development was conducted from March 2022 to June 2022 using PubMed, Google Scholar and Web of Science databases using keywords such as knowledge, attitude, practice, green dentistry, ecofriendly dentistry, and sustainable dentistry. Every individual search term was supplemented with relevant free text terms. When appropriate, the free text terms have been truncated in order to include alternative word endings. The database searches were complemented with manual review of the reference lists of relevant articles, which resulted in a few additional articles included in the study.

**Fig.1:**
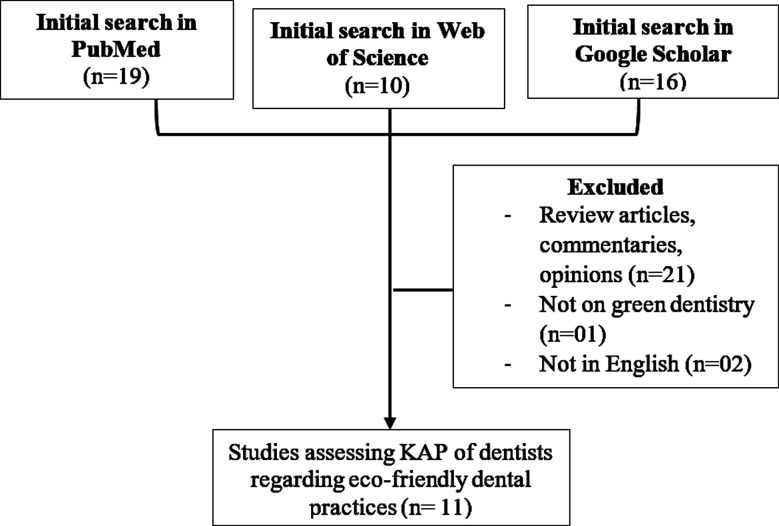
Flowchart of literature search.

Studies exploring knowledge, attitude, and practices of dentists regarding green dentistry in English with free full text were included. Thirteen articles were selected, of which two non-English were excluded. A manual screening of references from final articles was performed to ensure inclusion of all articles. The eleven studies were all original research manuscripts, with one being a thesis dissertation. Fig.1 shows the flowchart of the search strategy.

### Ethical approval

Not Applicable.

## RESULTS

Articles on KAP of dental health care providers regarding green dentistry are summarized in Table-I. All studies used a self-administered questionnaire as their data collection tool, with responses ranging from 150 to 800. The respondents were mostly dentists, dental graduate and post graduate students, and dental assistants from regions including Thailand, Romania, India, Jordan, and Saudi Arabia.

**Table-I:** Summary of final articles included in the review.

No.	Author	Study Type	Country	Participants	Sample size	Instrument	Publication year
1.	Agrasuta V^9^	Cross sectional KAP	Thailand	Dentists	472	50-item Questionnaire	2013
2.	Shatrat A^8^	Cross sectional KAP	Jordan	Dentists	150	39-item questionnaires	2013
3.	Zahrunnissa^10^	Cross sectional KAP	India	Dentists	150	22-item questionnaire	2015
4.	Daniela^26^	Cross sectional attitudes and behaviors	Romania	Dentistry students, dental practitioners, dental technician)	159	Questionnaire	2015
5.	Mohammad A. Al-Qarni^11^	Cross sectional pre-post survey knowledge	Saudi Arabia	Dentists, dental students, dental auxiliaries	160	18-item questionnaire	2016
6.	Prathima^13^	Cross sectional KAP	India	Dentists	800	Questionnaire	2017
7.	Amandeep^16^	Cross sectional practices and perceived barriers	India	Dentists	120	6-item questionnaire	2017
8.	Abhinav	Cross sectional attitudes and factors influencing adoption of green dentistry	India	Dentists	110	9-item questionnaire	2017
9.	Praneetha^15^	Cross sectional attitude and implementation of ecofriendly dental office strategies	India	Dentists	120	Questionnaire	2019
10.	Pallavi^12^	Cross-sectional KAP	India	Dentists	250	15-item Questionnaire	2019
11.	Shivangi^14^	Cross sectional knowledge and practices	India	Dentists	200	20-item questionnaire	2020

### Knowledge of green dentistry

Six of eleven articles reported dental health care providers’ knowledge or awareness of green dentistry, with Table-II providing a summary of studies. In 2013, two studies were conducted in Jordan and Thailand to assess the KAP of dentists regarding green dentistry using self-developed questionnaires.^8^,^9^ Shatrat et al. in Jordan tested questionnaire on 30 volunteer participants for content validity, clarity, and format.^8^ Agrasuta V’s study did pilot for clarity and ease of completion.^9^ Both studies reported no quantitative measures of internal validity or consistency and showing dentists are well-versed in sustainable dental practices. However, the design of the questionnaire, which forbade the use of parametric statistical tests, is one of the limitations of both studies that might have had an impact on the findings. Both studies expressed concern related to the lack of literature on eco-friendly dental practices, which also makes it difficult to validate the findings.

**Table-II:** Summary of studies assessing knowledge of dentists regarding green dentistry.

No.	Author	Country	Instrument	Knowledge assessment	Knowledge result	Key findings
1.	Shatrat A^8^	Jordan	45 item Inn person self-administered questionnaire, 4-point Likert scale.	4-point Likert scale Scale options: Fully in place. In progress Aware of strategy but not implemented	70% reported high knowledge	70% of dentists: high knowledge of eco-friendly dental practices. Highest knowledge observed: alternative filling materials to amalgam, amalgam disposal, utilizing and purchasing radiographic chemicals, and compact fluorescent light bulbs. Least knowledge: radiographic waste disposal and using motion detectors for rooms.
2.	Agrasuta V^9^	Thailand	Web based 7 multiple choice knowledge questions.	Assessed as scores. Highest score: 6 High: 4 to 5 moderate:3 low: 1 to 2 very low: 0	Score 5: 35.6% Score 4: 29.2 % score 3: 13.1 % Score 1 to 2: 5%	83.5% of respondents never heard about the term “green dentistry” and 16.5% having some idea. Responses differed significantly for age, years of working experience, and workplace. Higher knowledge scores: age above 31 with more work experience Least knowledgeable: private dental practitioners
3.	Zaharunnisa^10^	North Bangalore, India	Self-administered 18 item questionnaire, in person.	Assessed as scores, reported as category. Inadequate (<=50% score) Moderate (51-75% score) Adequate (>75% score)	62% dentists: moderate knowledge, 38%: adequate knowledge,	62% moderate knowledge 38% adequate knowledge. Dentists of age >40 years significantly higher knowledge as compared to those < 40 years old. Males had > knowledge than females, Postgraduate dentists > knowledge than graduate dentists.
4.	M. Al Qarni^11^	Saudi Arabia	Self-administered 18 item questionnaire	Mean knowledge score	dental student 29.33 (11.8) lecturer 34.92 (11.53) General dental practitioner 37.60 (12.12) Professor 36.63 (13.10) Dental auxiliary 43.81 (10.95)	Overall participants lacked knowledge about eco-friendly dental practices.
5.	Prathima^13^	Telangana, India	Self-administered questionnaire	Not specified		13.1% respondents aware: Eco-Friendly Dental practices association. 76% participants aware of dental practice effects on environment
	Pallavi^12^	Chennai, India	8-items questionnaire	Proportion of correct answers for each stem	Awareness of green dentistry among: Postgraduates 73.6% graduates 50%	Postgraduate’s awareness of green dentistry > than graduates. Graduates knowledge about digital radiography (100%). Awareness of mercury toxicity high in both groups
6.	Shivangi^14^	Bhopal, India	20 item self-administered questionnaire	Proportion of correct answers for each stem	Dentists’ knowledge regarding green dentistry insufficient	52.5% PGs, 48.4 % graduates and 81.5% PG students aware of the term “green dentistry”.

KAP of dentists in India and Saudi Arabia was assessed in 2015 and 2016. Zaharunnissa et al used a self-developed questionnaire to survey 150 dental practitioners. Pilot tested on thirty volunteers. The study found that dentists had adequate knowledge about sustainable dentistry, with “fully in place” being a proxy for high knowledge.^10^ Al Qarni et al in Saudi Arabia conducted an educational awareness intervention to increase awareness of eco-friendly dentistry.^11^ Using a self-administered questionnaire with internal consistency of 0.80-1.00. Unlike other studies by Shatrat et al^8^, Agrasuta V et al^9^, and Zahrunnisa et al^10^, this study did not report final knowledge levels by means of continuous or categorical score. Instead, proportions of dentists responding correctly to each answer were reported with results showing improved knowledge of eco-friendly dental practices in post-intervention test.^11^

Three Indian studies were reported between 2017 and 2020 to assess dental practitioners’ knowledge on sustainable dental practices. Prathima et al reported results from 800 dentist participants, Pallavi et al and Shivanji et al had 150 and 200 participants respectively.^12^-^14^ They used self-administered questionnaires.^14^ Dental practitioners’ knowledge of green dentistry was found to be high, while Shivangi et al reported otherwise.^12^ According to Pallavi et al the results cannot be regarded as having actual validity because the degrees of awareness in urban and rural areas of the country may differ.

### Attitudes about green dentistry

Seven of eleven studies reported positive dentists’ attitudes towards green dentistry, with each study reporting on diverse themes as summarized in Table-III. Overall, dentists have a positive attitude towards adopting green dental practices to reduce environmental impact. Two studies reported negative attitudes towards green dental practices due to their perceived financial burden.^9^,^15^

**Table-III:** Summary studies assessing attitudes regarding green dental practices of dentists.

No.	Author	Country	Instrument	Attitudes assessment	Attitudes result	Key findings
1.	Agrasuta V^9^	Thailand	11 questions	Assessed as scores on a 5-point Likert 1 = very negative 3 = neutral 5= very positive	Mean attitude score 3.95 (SD 0.43)	Overall attitude: very positive Lowest attitude scores: financial burden, ease of finding green products, and amalgam management. Strong sense of dental community role in environment protection. Highest positive attitude scores: Energy management and water management 4.44 and 4.47 Negative responses: added financial burden. Concerns: about using sterilizable instruments over disposables.
2.	Zaharunnisa^10^	North Bangalore, India	5 questions	Assessed as scores, reported as category. Inadequate (<=50% score) Moderate (51-75% score) Adequate (>75% score)	56.7% moderate attitudes score, 43.3% adequate attitude score	Overall, dentists had adequate attitudes towards adopting green dentistry Dentists of age < 39 years had highest adequate attitude scores (55.8%), Lowest scores > 40 years old. Males had highest attitudes than females. Graduate dentists had higher proportion of attitudes than PGs. Dentists with 10-15 years of experience had most positive attitudes (57.1%)
3.	Daniela^26^	Romania	Self-administered questionnaire	Proportions	Not specified, study was ongoing, presented partial results	Positive response towards biomedical waste management. Unaware of opportunities to recycle clinical waste
4.	Prathima^13^	Telangana, India	Self-administered questionnaire	Not specified	Not specified	91.9% positive attitudes towards implementing various green dental strategies.
5.	Praneetha^15^	Rajahmundri , India	8-items on a questionnaire	Measured as categories: Agree Not sure Disagree	Not specified	90% agreed that green practices play a role in environment 38.3% transformation from current to Green practice is difficult 63.3% unsure if green practices will increase financial burden 41.7% unsure of finding compatible green dentistry products 51.7% believed that eco-friendly practices lower operations and maintenance cost.

### Green dental practices

Eight studies assessed practices of dental health care providers on green dentistry using self-administered questionnaires with varying numbers of questions. The questionnaires focused on amalgam, radiographic, waste, water, and energy management. Shatrat et al and Agrasuta et al. reported green practices of dentists as being “fully implemented”, “in the process”, “not implemented” or “unsure”.^8^,^9^

Other studies reported proportion of dentists following each practice. Regardless of measurement, there was low implementation of sustainable dental practices by dental practitioners.^12^-^16^ Majority reported Dentists of different ages, genders, post-graduates, and under-graduates have different practices.^9^,^10^,^12^,^14^ Nevertheless, many studies in terms of evaluating green practices were not free from biases. According to Zahrunnisa et al, the questionnaire could not address all eco-friendly dentistry practices because it only included a few in order to encourage compliance which makes it harder to support the findings. 10 Similar to this, Al Qarni et al in Saudi Arabia focused on the role of dentists, but it might not have correctly represented the relevance of dental hygienists and assistants in the adoption of eco-friendly dentistry practices. 11 The issue of limited sample size was common with the most studies in India; hence, investigations with a broader population were required to have a deeper understanding and more trustworthy data to generalize the findings.^12^-^16^

Majority using GIC and composites conserve water and electricity.^10^,^13^,^15^,^16^ Amalgam separators were least used with poorly established amalgam disposal practices.^8^,^10^,^13^,^15^,^16^ Majority of dentists have digitalized their radiographs and record keeping systems. Chemicals are often purchased in concentrated, bulk form from vendors. Reducing waste, using renewable energy, and using eco-friendly paint are important strategies for infection prevention in different regions. A summary of findings for practices is shown in Table-IV.

**Table-IV:** Summary studies assessing green dental practices of dentists.

No.	Author	Country	Instrument	Practice assessment	Practice result	Key findings
1.	Agrasuta V^9^	Thailand	15-item self-administered online questionnaire	‘Fully implemented’ ‘In process’ ‘Not implemented’ ‘unsure’	47.3% adopting green practices (Low)	< 50% eco-friendly dental practices. Pre-capsulated amalgam usage (92.8%) in the public sector. Radiographic waste, and water management was less than 50%. General waste management, infection control management energy management was >50% but < 80%.
2.	Shatrat A^8^	Jordan	45-item self-administered questionnaire	‘Fully implemented’ ‘In process’ ‘Not implemented’ ‘unsure’	Low	Low: amalgam management, radiographic and paper waste management, electronic records, infection control management and water/energy conservation. High implementation: chemical mixing and storing, purchasing concentrated chemicals, digital radiography and fluorescent light bulbs.
3.	Zaharunnisa^10^	North Bangalore, India	13-items in a self-administered questionnaire	Practice measured as: Inadequate < or =50% Moderate 51- 75% Adequate > or = 75%	62% dentists moderate practice levels 38% adequate practice levels	71.3% implementation: electronic records & awareness videos, 99% use of towel, 56.6% recycling paper, and 73.4% using digital radiography. 94.7% sterilizable instruments rather than disposables, 80% compact fluorescent lights, 93.7% hand sanitizers, 88% bulk purchasing, and 74% segregation of lead foil. Low scores: unused amalgam particles in closed containers (43.3%) and amalgam separators (36.6%).
4.	Prathima^13^	Telangana, India	Self-administered questionnaire	Positive proportions for each practice	Low	60% practiced paper waste disposal and alternative filling materials to amalgam. Low implementation: other green dental practices -renewable energy, waste segregation, digital data & radiography, recycling x-ray fixer, developer, & lead foil, and using amalgam separators.
5.	Amandeep^16^	Chandigarh, India	6-item questionnaire	Positive proportions for each practice	Implementation in its stage of infancy	> 90% using alternate filling materials, LED bulbs, steam sterilizable cloth wraps. 78% digital radiography
6.	Praneetha^15^	South Indian state, India	60-item questionnaire	Positive proportions for each practice	Adequate	81.7% use alternatives to amalgam fillings, 91.7% use digital radiographs, 48.3% use sterilizable instruments, 45% use LED light bulbs, 66.7% used hand sanitizers.
7.	Pallavi^12^	Chennai, India	12-item questionnaire	Positive proportions for each practice	Inadequate 45.3% PGs and 29.4% graduates	83.9% used LED bulbs in their clinics, 37.9% used green plants to increase oxygenation, 52.9% digital record, 69% used disposable drapes, 90.8% plastic suction tips. 62.3% digital radiography (PG practitioners) v/s 35.5% graduate practitioners.
8.	Shivangi^14^	Bhopal, India	10-items in a self-administered questionnaire	Positive proportions for each practice	Not up to mark.	GIC and composite as an alternate to amalgam fillings Majority respondents (>50%) recycled x-ray fixer and developer solution, reusable lab coats and patient drapes, LED light bulbs and dry dental vacuum pump. Varying mercury disposing practices, majority disposing mercury in sink. Majority practicing conservation of water and electricity.

## DISCUSSION

Green dentistry seeks to promote sustainability in dental practice. Farahani and Suchak pioneered eco-friendly dentistry in 2007.^3^ Their green model of eco-friendly alternatives to conventional dental practices replaced harmful current dental practices with green practices.^3^ Since then, numerous studies have explored the environmental footprint of dental practices.

The review found that the lack of literature on eco-friendly practices in dentistry made it difficult to validate the findings of most of these studies. This led to the absence of a uniform instrument, which is pervasive throughout literature and is identified as a common barrier. The six green dentistry model themes—amalgam management, radiography management, infection control management, procurement management, energy and water conservation—were shared by all of them. Despite common themes, each study had developed their own tool. Overall, most studies reported high dentists’ knowledge of green dental practices, but their practices were not adequate.

### Amalgam management

Regarding the first theme, amalgam management, its usage is contentious because of mercury, a bio-accumulating substance.^17^ Mercury is known to have negative effects on human health.^18^,^19^ Improper disposal of amalgam in regular trash is hazardous. Unused amalgam particles should be collected and recycled, and pre-capsulated amalgam should be used. Amalgam separators should be installed in suction and vacuum lines.^3^,^4^,^20^ All studies examined the environmental effects of amalgam and mercury, and asked questions about dentists’ knowledge and practice. Respondents in Jordan, Thailand, Saudi Arabia, and India were aware of amalgam hazards.^8^,^9^,^11^,^12^,^14^,^15^ Dentists in Jordan, Thailand, and India used alternative filling materials to replace amalgam. However, the use of amalgam separators was low due to dentists disposing the amalgam in garbage and the lack of proper disposal practices with few using amalgam separators. Amalgam is a popular restorative material due to its affordability, longevity, and ease of use among Indian dentists.^21^,^22^

### Radiography management

Conventional radiographs use x-ray fixer, developer and dental film, which contain hazardous chemicals and should not be disposed of in regular trash.^8^,^23^ Dental practitioners must be aware of hazardous components and proper disposal of conventional x-rays. Green dentistry recommends digital radiographic techniques to minimize harmful chemical, paper, and plastic waste.^24^ Majority of dental practitioners in Jordan and North Bangalore use digital radiographs, while low levels of usage are reported in Chennai and Thailand.^8^-^10^,^12^ Poor disposal of x-ray fixer and developer solutions is observed.^8^-^10^,^12^

### Infection control management

Only 20% medical waste is considered infectious and requires special disposal, while 3% from a dental practice requires management with protocol.^25^ Studies included in this article reported high knowledge of eco-friendly infection control strategies.^8^-^14^,^16^,^26^,^27^ Sterilizing instruments, film holding devices and trays, are the most common green infection control practices. Amandeep et al reported 91% dentists use steam sterilization for disinfection, while other studies reported low implementation.^16^ Enzyme-based cleaners are biodegradable and free of harsh chemicals, making them ideal for green practices.^4^,^24^,^28^Practices of using enzyme-based cleaners and reusable items are low, with more inclination towards disposable items^4^,^24^,^28^. Eco-friendly dental recommendations include paper recycling, electronic records, water and electricity conservation.^3^,^24^ Technology and awareness of recycling programs are essential for successful use of electronic records. Dentists in Jordan and Thailand recycle and use e-records. In contrast paper waste management was not well practiced in India, as reported by Praneetha et al and Pallavi et al.^12^,^15^

The convergence of views regarding many facets of green dentistry is one of the review’s main conclusions. The majority have a positive attitude towards environmental protection. This can be used to increase dental practitioner’s engagement in sustainable practices by creating awareness. However, affordability, adaptation, and availability of green dental products are linked to negative attitudes. Green dental products are expensive and limited, making it difficult to adopt sustainable practices.^29^ Additionally, altering conventional dental practices necessitates a gradual transformation in thinking and behaviour.^30^ This study reviews the literature on dentists’ KAP with regard to clinical green dental practices. It highlights the need for more stringent research on the subject and create a standardized tool to assess KAP before developing interventions to improve it.

### Limitations

The study scope is limited to only original KAP studies.

## CONCLUSION

The practice of dentistry has a significant environmental impact due to its use of raw materials, water, and electricity. The studies identified in this paper provide a heightened level of knowledge, attitude and practices and serves as a rigorous review of literature pertaining to studies done globally for exploring dentists’ KAP regarding green dental practices. Although dental professionals are aware of eco-friendly practices and have a positive attitude, their practice is far from acceptable. The greatest barrier to the implementation of sustainability in all studies is discernment that exists within the profession that do not consider or prioritize sustainable practices. These are particularly pervasive in developing countries, where the greatest barriers are a lack of economic feasibility and a poor knowledge-base on the subject. The lack of literature on eco-friendly practices in dentistry makes it difficult to validate the findings of the majority of these studies. This has led to the absence of a uniform instrument, which is ubiquitous throughout literature and is identified as a common barrier. There is a notable dearth of quality research that encourages and makes it possible to provide environmentally sustainable oral health care. Emerging research should attempt to develop a standardized tool comprising common aspects of green dentistry, to measure KAP of dentists, prior to planning interventions for improvement.

### Author’s Contributions:

**JGD, JJ** and **NZ:** Conceived the project, format, the main conceptual ideas, and proof outline.

**JGD:** Is responsible for accuracy and integrity of the work.

**JGD** and **JJ:** Conceived the study and oversaw overall direction and planning.

**NZ:** Wrote the manuscript with support from **JGD**, **JJ** and **JP** who analyzed the data.
